# Editorial: Recent Advancements in Structural Equation Modeling (SEM): From Both Methodological and Application Perspectives

**DOI:** 10.3389/fpsyg.2018.01936

**Published:** 2018-10-09

**Authors:** Oi-Man Kwok, Mike W. L. Cheung, Suzanne Jak, Ehri Ryu, Jiun-Yu Wu

**Affiliations:** ^1^Texas A&M University, College Station, TX, United States; ^2^National University of Singapore, Singapore, Singapore; ^3^University of Amsterdam, Amsterdam, Netherlands; ^4^Boston College, Chestnut Hill, MA, United States; ^5^National Chiao Tung University, Hsinchu, Taiwan

**Keywords:** structural equating modeling, measurement invariance (MI), multilevel (ML), Mediation, meta analysis, effect size, complex data

Structural equation modeling (SEM) is becoming the central and most popular analytical tool in the social sciences. Many classical and modern statistical techniques such as regression analysis, path analysis, confirmatory factor analysis, and models with both measurement and structural components have been shown to fall under the umbrella of SEM. Thus, the flexibility of SEM makes it applicable to many research designs, including experimental and non-experimental data, cross-sectional and longitudinal data, and multiple-group and multilevel data. Further enhancing the popularity and widespread use of SEM, it has recently experienced exciting advancements—from fundamental issues like alternative estimation methods that are robust to often violated assumptions to the expansion of SEM to incorporate multilevel and cross-classified data that are common in the social sciences. This Special Research Topic aims to bring in a collection of SEM papers that not only tackle technical estimation issues but also examine and demonstrate application of SEM to more complex settings, such as applying robust estimation method, testing interaction effect, examining measurement invariance, and specifying and evaluating models applied to different types of data, including meta-analytic data, multilevel, and longitudinal data.

We are presenting 19 cutting-edge papers covering a wide variety of topics related to SEM. The papers have been grouped into three main themes: (a) analysis of different types of data (from cross-sectional data with floor effects to complex survey data and longitudinal data); (b) measurement-related issues (from the development of new scale to the evaluation of person fit and new ways to test measurement invariance); and (c) technical advancement and software development. Below you will find a summary of the three themes and corresponding papers for each.

## Analysis of different types of data

One of the major advantages of SEM is its flexibility for analyzing different types of data. On this research topic, Zhu and Gonzalez have demonstrated how to analyze multilevel data with strong floor effects using multilevel SEM and examined the impact of ignoring these floor effects when using regular multilevel analysis via a Monte Carlo study. Similarly, Wu et al. have demonstrated the multilevel confirmatory factor analysis with the use of complex survey data and compared different approaches to analyzing this type of data. Their simulation results showed that the maximum modeling strategy generally outperforms the other approaches.

In addition, several papers focus on the analysis of longitudinal data from different perspectives. Ning and Luo have introduced and evaluated a new piecewise growth-curve model (PGCM) without the requirement of pre-specifying the turning point. Similarly, Kim et al. have proposed an optimal starting model under the latent growth modeling (LGM) framework when searching for the accurate growth trajectory. These authors found that the fully saturated model performed the best even with the presence of the time-invariant covariates in the LGM (i.e., the conditional LGM). Kamata et al. have investigated the performance of three approaches (i.e., one-step, three-step, and case-weight) on estimating a two-phase mixture model with an auxiliary linear-growth model. This simulation study showed that under different conditions some approaches outperformed the others (e.g., both case-weight and three-step resulted in higher convergence rate but could also lead to substantially underestimated standard errors when the class separation was low). As an extension of the mixture model, the growth-mixture model (GMM) is another commonly used model for analyzing longitudinal data. Focusing on GMM, Kim and Wang have conducted two Monte Carlo studies to examine the impact of ignoring the presence of measurement non-invariance between latent classes in terms of class enumeration and parameter recovery when applying both GMM and second-order GMM. In general, the second-order GMM outperformed the traditional GMM with more accurate class enumeration and unbiased parameter estimates. For more complex longitudinal data such as students moving to different classrooms over time, Kwok et al. have demonstrated how to analyze this type of data, especially in terms of capturing the carry-over effect, with the use of the Project ELLA data along with the xxM program.

## Measurement-related issues

Measurement models are an important part of SEM, and the flexibility of SEM not only allows researchers to develop and validate new scales but also provides a simple and feasible platform for examining the potential differences between groups and populations through the test of measurement invariance. Zhao et al. have developed and validated their online shopping addiction scale with both exploratory factor analysis (EFA) and confirmatory factor analysis (CFA). Similarly, Glaman and Chen have tested the measurement invariance of a classroom engagement measure among academically at-risk students across grades, genders, and ethnicities. In addition, several excellent papers address the methodological issues involved in testing measurement invariances, including Jorgensen's work  on using permutation tests and multivariate modification indices, Jiang et al.'s study on using equivalence tests with a projection-based approach on testing measurement invariance and mean comparison, and Hsiao and Lai 's paper on examining the impact of partial measurement invariance on testing moderation for single and multilevel data.

By extending the measurement test to more complex data settings, Jak and Jorgensen have showed the relationship between measurement invariance, cross-level invariance, and multilevel reliability. Moreover, Guenole has evaluated the impact of biased-referent indicators with the use of free vs. constrained baseline approaches within a multilevel SEM framework. His simulation results re-emphasize the importance of having an unbiased referent indicator when testing measurement invariance.

## Technical advancement and software development

This special research topic includes several articles on new technical advancement and software development in SEM. For example, effect size reporting becomes necessary and is required by most of the prestigious peer-reviewed journals in behavioral and social sciences. Cheung has addressed the importance of the multivariate effect sizes and demonstrated how to compute multivariate effect sizes and the corresponding covariance matrices under the SEM framework with the use of the metaSEM package.

The normal-theory maximum likelihood (ML-Normal) is the most commonly used estimation method in SEM and is also the default estimation method for most SEM-related software. However, ML-Normal is not efficient and can be severely biased by outliers and influential observations in the data. Lai and Zhang have evaluated the performance of the fit indices from the multivariate *t*-based SEM framework and recommend that the multivariate *t*-based SEM be used when outliers and influential observations exist in the data. Similarly, given that linear factor analysis (FA) is another commonly used SEM approach for psychometric applications, Ferrando et al. have proposed a simple and workable approach that can routinely assess person fit in FA-based studies. Through both simulation study and real-data demonstration, they found that the mean-squared *lico* index and the personal correlation work well in conjunction and can function effectively for detecting different types of inconsistency.

Mediation or indirect effect is fundamental to many substantive areas. For comparing indirect effects in different groups, Ryu and Cheong have examined both single-group and multiple-group SEM approaches, concluding that the multiple-group approach is generally the preferable approach. They also recommend the use of the bootstrap confidence intervals when adopting the single-group approach. In a similar vein, confirmatory factor analysis (CFA) is commonly used in the social sciences, but specifying the model is sometimes tricky, especially for complex data settings such as multilevel data. Hence, Wu et al. have developed an integrated MCFA (iMCFA) program that allows researchers to easily and flexibly fit the single-level CFA models as well as the multilevel CFA models with maximum model at either the within- or the between-level.

We hope that the readers will gain new perspectives and be able to apply some of the new techniques and models discussed in the 19 papers in this special research topic. Moreover, we hope that more advanced readers will conduct more exciting studies and move the field by extending some of the methods and ideas from the papers in this special topic.

## Author contributions

All authors listed have made a substantial, direct and intellectual contribution to the work, and approved it for publication.

### Conflict of interest statement

The authors declare that the research was conducted in the absence of any commercial or financial relationships that could be construed as a potential conflict of interest.

